# Iron Promotes Cardiac Doxorubicin Retention and Toxicity Through Downregulation of the Mitochondrial Exporter ABCB8

**DOI:** 10.3389/fphar.2022.817951

**Published:** 2022-03-11

**Authors:** Archita Venugopal Menon, Jonghan Kim

**Affiliations:** ^1^ Department of Pharmaceutical Sciences, Northeastern University, Boston, MA, United States; ^2^ Department of Biomedical and Nutritional Sciences, University of Massachusetts Lowell, Lowell, MA, United States

**Keywords:** cardiotoxicity, doxorubicin efflux, hemochromatosis, iron chelator, iron overload

## Abstract

In several cancers, the efflux and resistance against doxorubicin (DOX), an effective anticancer drug, are associated with cellular iron deficiency and overexpression of the mitochondrial exporter ABCB8. Conversely, decreased ABCB8 expression and disrupted iron homeostasis in the heart have been implicated in DOX-associated cardiotoxicity. While studies have demonstrated that altered iron status can modulate the susceptibility to DOX cardiotoxicity, the exact molecular mechanisms have not been clearly understood. Here, we hypothesized that iron stores influence cardiac ABCB8 expression and consequently cardiac retention and toxicity of DOX. First, we found that ABCB8 deficiency in cardiomyocytes decreased DOX efflux, increased DOX-induced toxicity, and decreased cell viability. Conversely, intracellular DOX retention and toxicity were ameliorated by ABCB8 overexpression. To determine if altered cardiac iron status modifies ABCB8 expression, we treated cardiomyocytes with high iron or iron chelators. Western blot and qPCR analyses revealed that ABCB8 levels were decreased in iron overload and increased in iron deficiency. Subsequently, DOX retention and toxicity were increased in cardiomyocytes with iron overload, whereas iron deficiency ameliorated these effects. Next, we validated our results using a mouse model of hereditary hemochromatosis (HH), a genetic iron overload disorder. HH mice exhibited decreased ABCB8 expression and increased DOX retention and toxicity. These changes were abolished by the treatment of HH mice with a low-iron diet. Finally, cardiac-specific overexpression of ABCB8 in HH mice prevented cardiac DOX accumulation and abrogated DOX-induced cardiotoxicity without altering iron overload in the heart. Together, our results demonstrate that ABCB8 mediates DOX efflux and that iron regulates DOX retention and toxicity by altering cardiac ABCB8 expression. Our study identifies a novel role of iron in DOX-induced cardiotoxicity and suggests potential therapeutic intervention for DOX and anthracycline-based cancer pharmacology.

## Introduction

Although doxorubicin (DOX) is an effective anticancer agent, it is non-selective towards cells and thus accumulates in other organs, primarily the heart, exerting off-target toxicities. Furthermore, DOX has a high affinity for the mitochondrial phospholipid, cardiolipin and preferentially localizes in the mitochondria (de Wolf et al., 1990; Ichikawa et al., 2014). Thus, the heart, rich in mitochondria, is particularly susceptible to the toxic effects of DOX.

The expression of cellular and subcellular drug transporters is a key determinant in the sensitivity and toxicity of anticancer agents, like DOX. For example, it has been known that ABC (ATP-binding cassette) protein expression significantly correlates with chemosensitivity in cancer (Clarke et al., 2005; Noguchi et al., 2014). These proteins (summarized in (Mohammad et al., 2018)) are active transporters which utilize ATP to translocate substrates across cellular and subcellular membranes and have been extensively studied since their overexpression is linked to increased cellular DOX efflux and resistance in several cancers (Szakács et al., 2006; Martin et al., 2014). Primarily, ABCB (particularly ABCB1), ABCC, and ABCG subfamilies have been linked to DOX resistance (Borst et al., 2000; Szakács et al., 2006; Fletcher et al., 2010; Bao et al., 2011). However, two major concerns exist to therapeutically target the currently known DOX efflux transporters: 1) the expression patterns of these ABCB proteins are heterogenous across different cancers (Szakács et al., 2004; Morton et al., 2019) and 2) efforts to modulate the expression of these transporters using small molecules, while efficacious in reversing DOX resistance, exacerbate DOX cardiotoxicity (Akimoto et al., 1993; Zhou et al., 2016). Hence, there is a need to identify novel DOX efflux transporters to efficiently correct DOX-induced cardiotoxicity.

ABCB6, ABCB7, ABCB8, and ABCB10 are the four currently known mitochondrial ABCB proteins and are all implicated in mitochondrial iron homeostasis (Zutz et al., 2009). However, the cardiac expression of these ABCB proteins, except ABCB8, is unchanged following DOX exposure (Ichikawa et al., 2014; Morton et al., 2019). ABCB8 is located in the inner mitochondrial membrane and has been implicated in exporting potassium, peptides, and iron from the mitochondria although the exact transport mechanism remains to be established (Ardehali et al., 2005; Ichikawa et al., 2012; Mohammad et al., 2018; Paggio et al., 2019). Studies suggest that, in addition to recognized substrates, ABCB8 may also efflux DOX; Mouli et al. demonstrated that frataxin deficiency is correlated with decreased ABCB8 expression and increased cellular DOX retention (Mouli et al., 2015). Wen et al. reported that inhibition of ABCB8 using a small molecule reversed DOX resistance by increasing DOX retention in breast cancer cells (Wen et al., 2018). However, Ichikawa et al. suggested that ABCB8 modulates the sensitivity to DOX cardiotoxicity through regulation of mitochondrial iron levels (Ichikawa et al., 2014). Interestingly, DOX cardiotoxicity is associated with *cardiac* iron accumulation and dose-dependent *depletion* of ABCB8 (Miranda et al., 2003; Ichikawa et al., 2014; Mouli et al., 2015), whereas DOX resistance in *cancer* is commonly associated with ABCB8 *overexpression* and unchanged or deficient cellular iron (Elliott and Al-Hajj, 2009; Chekhun et al., 2013; Basu et al., 2017; Bajbouj et al., 2019). These results prompted us to hypothesize that cellular iron status modulates cardiac ABCB8 expression and consequently cardiac retention and toxicity of DOX. We tested our hypothesis using cardiomyocytes with altered iron and ABCB8 levels and verified our results *in vivo* using mice with hereditary hemochromatosis (HH), a genetic iron overload disorder associated with exacerbated DOX cardiotoxicity in humans (Zhou et al., 1998; Lipshultz et al., 2013). Our study cumulatively demonstrates that iron aggravates cardiac DOX retention and toxicity through downregulation of the DOX exporter ABCB8.

## Materials and Methods

### Cell Culture and Drug Treatment

H9C2 rat cardiomyoblasts (ATCC) were pretreated with ferric ammonium citrate (FAC, 100 µg iron/ml) or the iron chelator deferoxamine (DFO; 200 μM) for 48 h followed by co-treatment with DOX (10 μM) for 24 h. For knockdown (KD) or overexpression (OE) of ABCB8, H9C2 cells were transfected with siRNA (Qiagen) or plasmid DNA (Origene Technologies), respectively, for 48 h, followed by DOX treatment for an additional 24 h.

### MTT Assay

Cell viability was determined using (3,4,5-dimethylthiazol-2-yl)-2,5-diphenyltetrazolium bromide (MTT, Sigma). MTT assay was performed by adding 100 μl of MTT (0.5 mg/ml) in complete media to each well and incubated for 2 h. Media was aspirated and 100 μl of DMSO was added to each well to solubilize the formazan crystals and absorbance was determined at 570 nm.

### DOX Efflux Assay

ABCB8-KD and ABCB8-OE cells were incubated with DOX (30 µM) for 3 h. Cells were then washed with HBSS, and media was replaced with DOX-free media and cellular DOX concentrations were determined using fluorescence microscopy (Ex/Em: 488/560 nm) and normalized to Hoechst fluorescence (nuclear marker) at 0, 1, 2, 4, and 6 h after DOX was removed. Fluorescence from three randomly chosen fields was measured for each time point. Images were edited for background correction, contrast, and brightness similarly using ImageJ and Keyence BZX Analyzer. To determine the effects of iron on DOX retention, H9C2 cells were treated with FAC (100 µg iron/ml) or DFO (200 µM) for 48 h followed by co-treatment with DOX (30 µM) for 3 h. Cells were then washed with HBSS and replaced with DOX-free media containing FAC or DFO. DOX fluorescence was measured in live cells using fluorescence microscopy at 0, 1, 2, 4, and 6 h. For each time point, fluorescence was measured from three randomly chosen fields.

### *In Vivo* Acute DOX Cardiotoxicity

Hfe^−/−^ mice (male, 9 week-old), a mouse model of hereditary hemochromatosis (Zhou et al., 1998), and their age-matched wild-type control Hfe^+/+^ mice (129/SvJ background) were injected intraperitoneally with DOX (20 mg/kg) or saline and euthanized 4 days post-dose.

### Iron-Deficient Diet

Male Hfe^+/+^ and Hfe^−/−^ mice were fed iron-deficient diet (5 mg iron/kg diet) starting at 8 weeks of age. Serum iron levels were monitored weekly. When serum iron levels in Hfe^−/−^ mice on ID diet matched those in Hfe^+/+^ mice on facility chow (∼8 weeks), DOX (20 mg/kg) was administered as a single intraperitoneal injection, followed by euthanasia 4 days post-dose.

### *In Vitro* mRNA Transcription and Intracardiac mRNA Injection

mRNA was transcribed *in vitro* from plasmid DNA (Origene) using the MEGAscript™ T7 Transcription Kit (Thermo Fisher). mRNA (15 µg) in a saline citrate buffer was injected into the myocardium of Hfe^+/+^ and Hfe^−/−^ mice (male, 9 week-old). Mice were injected with DOX (20 mg/kg) 6 h after the mRNA injection and euthanized 4 days later.

### Western Blot Analysis

Proteins were extracted from hearts or cells using RIPA and separated using SDS-PAGE (20–30 μg protein) and transferred to PVDF membranes. Membranes were blocked with 5% nonfat milk and incubated overnight at 4°C with primary antibodies: ABCB8 (a kind gift from Dr. Hossein Ardehali, 1:2000), caspase-3 (Abcam, 1:500), or tubulin (Abcam, 1:2000). Membranes were incubated for 3 h at room temperature with anti-rabbit HRP-conjugated secondary antibody (Abcam, 1:2000). Protein expression was normalized to that of tubulin.

### Measurement of Iron

Hearts were digested in an acid solution (10% trichloroacetic acid and 3 M HCl) for 20 h at 65°C. Non-heme iron levels were measured by a colorimetric assay using bathophenanthroline disulfate (BPS) (Torrance and Bothwell, 1968). Mitochondria were isolated from hearts as previously described (Frezza et al., 2007) and suspended in 10 mM 2-(N-morpholino) ethanesulfonic acid (MES) and 1% SDS. Mitochondrial NHI levels were measured using BPS. Cells were washed twice with HBSS and incubated with calcein-AM and rhodamine B-[(1,10-phenanthroline-5-yl) aminocarbonyl] benzyl ester (RPA, 1 µM) for 20 min to determine cytosolic and mitochondrial labile iron levels, respectively (Petrat et al., 2002; Sturm et al., 2003). Fluorescence was determined by microscopy and normalized to Hoechst fluorescence. Heart labile iron levels were determined using dihydrorhodamine as previously described (Ye et al., 2019).

### Measurement of DOX in Heart

Mitochondria and cytosol were isolated as described previously (Frezza et al., 2007). Mitochondria were suspended in PBS and DOX measurements were measured using a fluorescence detector (Ex/Em: 470/560 nm). DOX concentrations were determined using a standard curve and normalized to protein content.

### Creatine Kinase-MB Analysis

Serum was obtained by centrifuging blood at 1,200 *g* for 12 min at room temperature. Serum CK-MB levels were measured using a commercial kit (MyBioSource) as per manufacturer’s instructions (Luu et al., 2020; Zhao et al., 2020).

### Statistical Analyses

Values were presented as mean ± SEM. Statistical significance was determined by the Student’s *t*-test for two-group comparisons or ANOVA with Tukey’s post-hoc comparisons for 4-group comparisons. Differences were considered significant at *p* < .05.

## Results

### ABCB8 Exports DOX out of Mitochondria and Decreases DOX Cardiotoxicity

**FIGURE 1:**
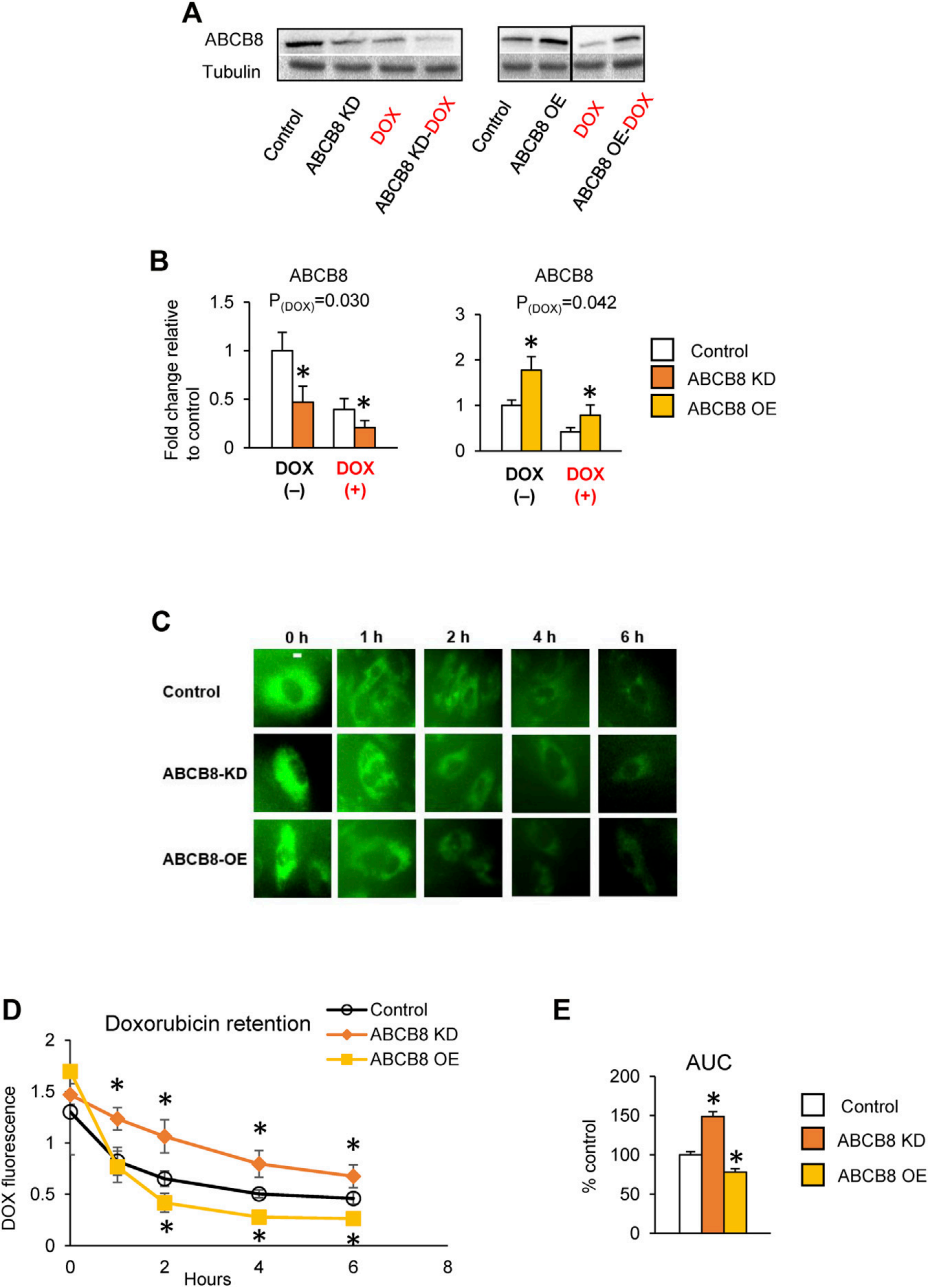
ABCB8 effluxes DOX from cardiomyocytes. **(A,B)** H9C2 cells were treated with ABCB8 siRNA or plasmid DNA and DOX (10 µM) as described in the Methods section. ABCB8 protein expression was determined using western blot and normalized to *α*-tubulin levels. Discontinuities between non-adjacent lanes of the same membrane were indicated by a solid line. **(C–E)** ABCB8 KD and ABCB8 OE cells were incubated with DOX (30 µM) for 3 h and DOX fluorescence was measured at the indicated times. Results are representative of four independent experiments **(C)**. DOX fluorescence was quantified over time **(D)**. The area under the curve (AUC) was calculated **(E)** from the DOX retention plot **(D)**. Data were expressed as mean ± SEM. Statistical significance was assessed using the Student’s *t*-test or ANOVA with Tukey’s post-hoc comparisons. *p < .05 vs. control. Scale bar = 10 µm.

To determine the effects of ABCB8 on cardiac DOX transport, we first generated ABCB8-deficient (ABCB8-KD, Supplementary Figures S1A,B) and ABCB8-overexpressing (ABCB8-OE, Supplementary Figures S1C,D) H9C2 cardiomyocytes and then treated the cells with DOX. In naïve H9C2 cells, DOX exposure resulted in decreased ABCB8 expression (Figures 1A,B). As predicted, DOX-induced downregulation of ABCB8 was exacerbated in ABCB8-KD cells and corrected by ABCB8-OE (Figures 1A,B). While ABCB8 is associated with DOX efflux and resistance in several cancers (Elliott and Al-Hajj, 2009; Basu et al., 2017; Wen et al., 2018), its role in cardiac DOX efflux remains to be established. To address this question, we altered ABCB8 expression in cardiomyocytes and determined DOX retention. While there were no differences in the initial cellular uptake of DOX (i.e., at 0 h) between ABCB8-KD or ABCB8-OE cells, intracellular DOX retention in ABCB8-KD cells was significantly greater compared with control cells at 1 h and onwards (Figures 1C,D). Conversely, DOX retention was significantly lower in ABCB8-OE cells (Figures 1C,D). Consistently, the total amount of drug retained in cardiomyocytes over 6 h was increased in ABCB-KD and reduced in ABCB8-OE cells (Figure 1E). These results indicate that ABCB8 plays an important role in cardiac DOX efflux.

**FIGURE 2:**
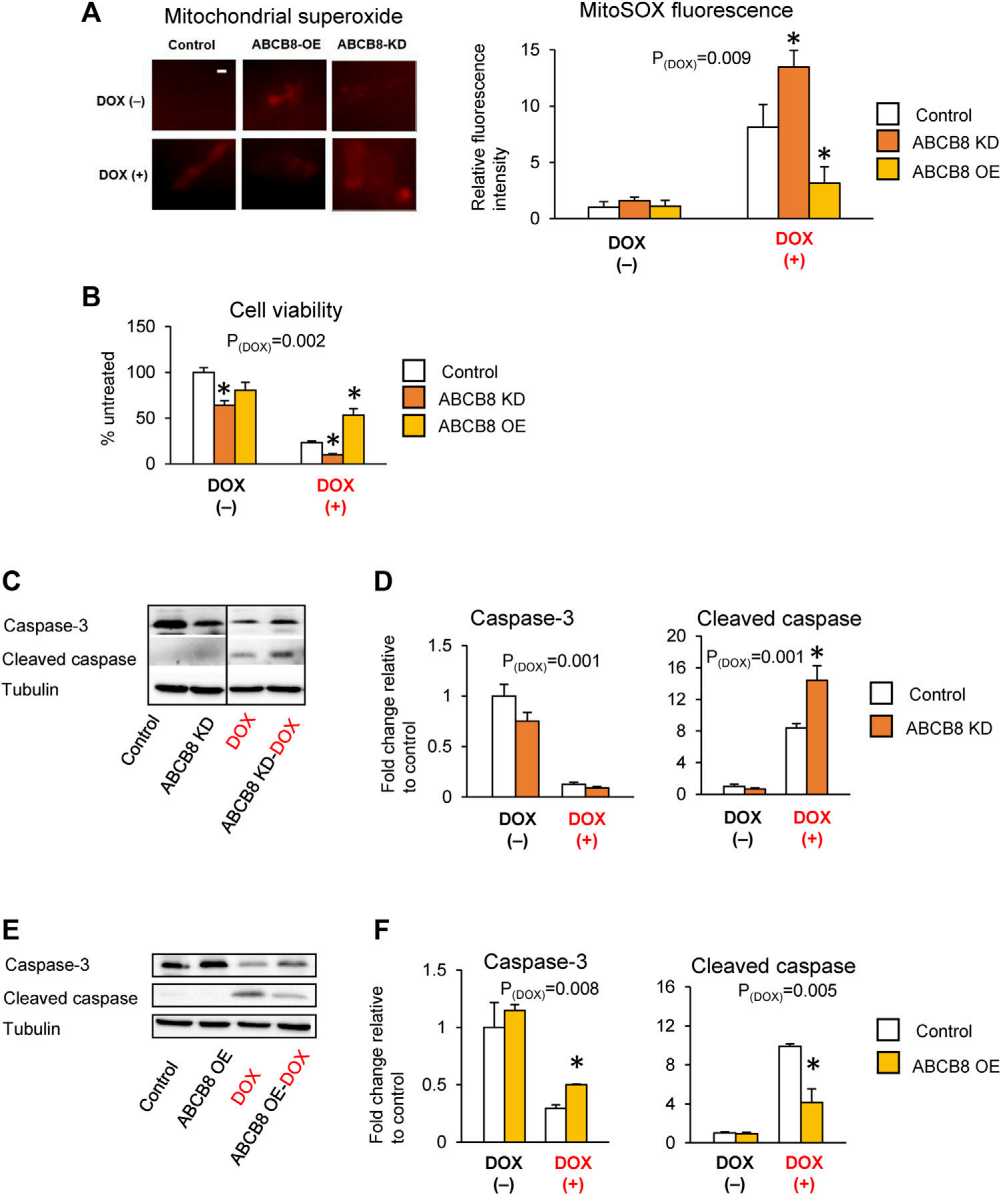
ABCB8 expression negatively influences DOX cardiotoxicity. H9C2 cells were treated with ABCB8 siRNA or plasmid DNA and DOX (10 µM) as described in the Methods section. ROS levels were analyzed by MitoSOX fluorescence **(A)**. Cell viability was determined using the MTT assay **(B)**. Caspase-3 and cleaved caspase levels were determined by western blot analysis **(C–F)**. Discontinuities between non-adjacent lanes of the same membrane were indicated by a solid line. Data were expressed as mean ± SEM. Statistical significance was assessed using the Student’s *t*-test or ANOVA with Tukey’s post-hoc comparisons. *p < .05 vs. control. Scale bar = 10 µm.

Next, we determined if altered ABCB8 expression influences DOX-induced cardiotoxicity. DOX-mediated oxidative stress (Figure 2A) and cardiac cell death (Figures 2B–F) were worsened in ABCB8-KD cells and protected in ABCB8-OE cells. Since DOX exposure is associated with cardiac mitochondrial iron overload (Ichikawa et al., 2014), we then examined the intracellular distribution of iron. Interestingly, mitochondrial iron in ABCB8-KD cells was insignificantly increased, whereas ABCB8 overexpression decreased mitochondrial iron (Supplementary Figure S1E). However, altered ABCB8 expression did not affect cytosolic labile iron with or without DOX (Supplementary Figure S1F). Thus, while the correlation between ABCB8 expression and iron levels in the mitochondria and cytosol remain unclear, our results suggest that ABCB8 primarily decreases the sensitivity to DOX cardiotoxicity by promoting DOX efflux from cardiac mitochondria.

### Iron Promotes Cardiac DOX Retention and Toxicity

**FIGURE 3:**
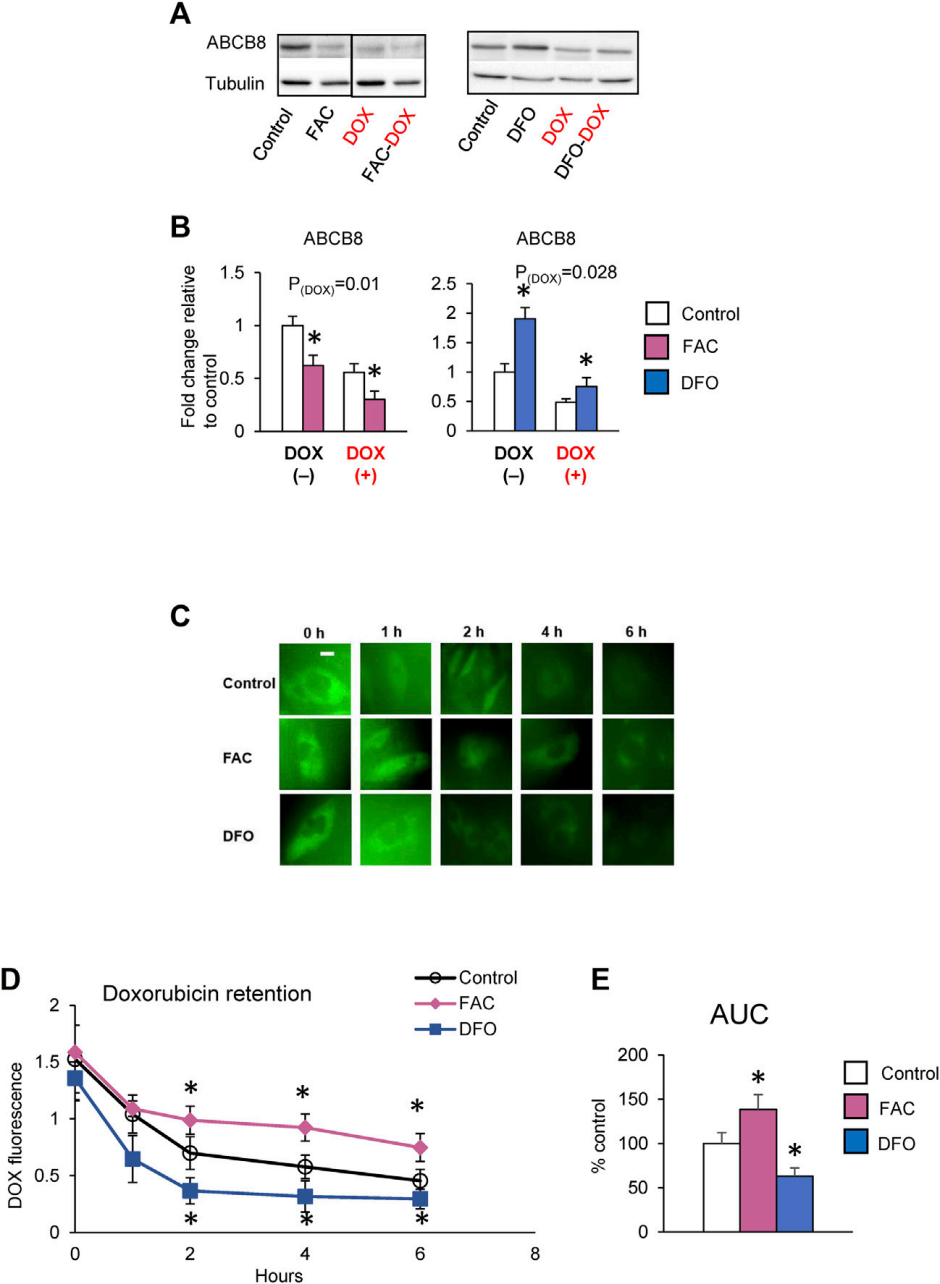
High iron promotes cardiac DOX retention. **(A,B)** H9C2 cells were treated with ferric ammonium citrate (FAC) or deferoxamine (DFO) and DOX (10 µM) as indicated in the Methods section. ABCB8 protein expression was determined by western blot analysis and normalized to *α*-tubulin levels. Discontinuities between non-adjacent lanes of the same membrane were indicated by a solid line. **(C–E)** After 48 h of FAC or DFO treatment, cells were incubated with DOX (30 µM) for 3 h DOX fluorescence was measured at the indicated times. Results are representative of four independent experiments **(C)**. DOX fluorescence was quantified over time **(D)**. AUC was determined from the DOX retention plot **(E)**. Data were expressed as mean ± SEM. Statistical significance was assessed using the Student’s *t*-test or ANOVA with Tukey’s post-hoc comparisons. *p < .05 vs. control. Scale bar = 10 µm.

**FIGURE 4:**
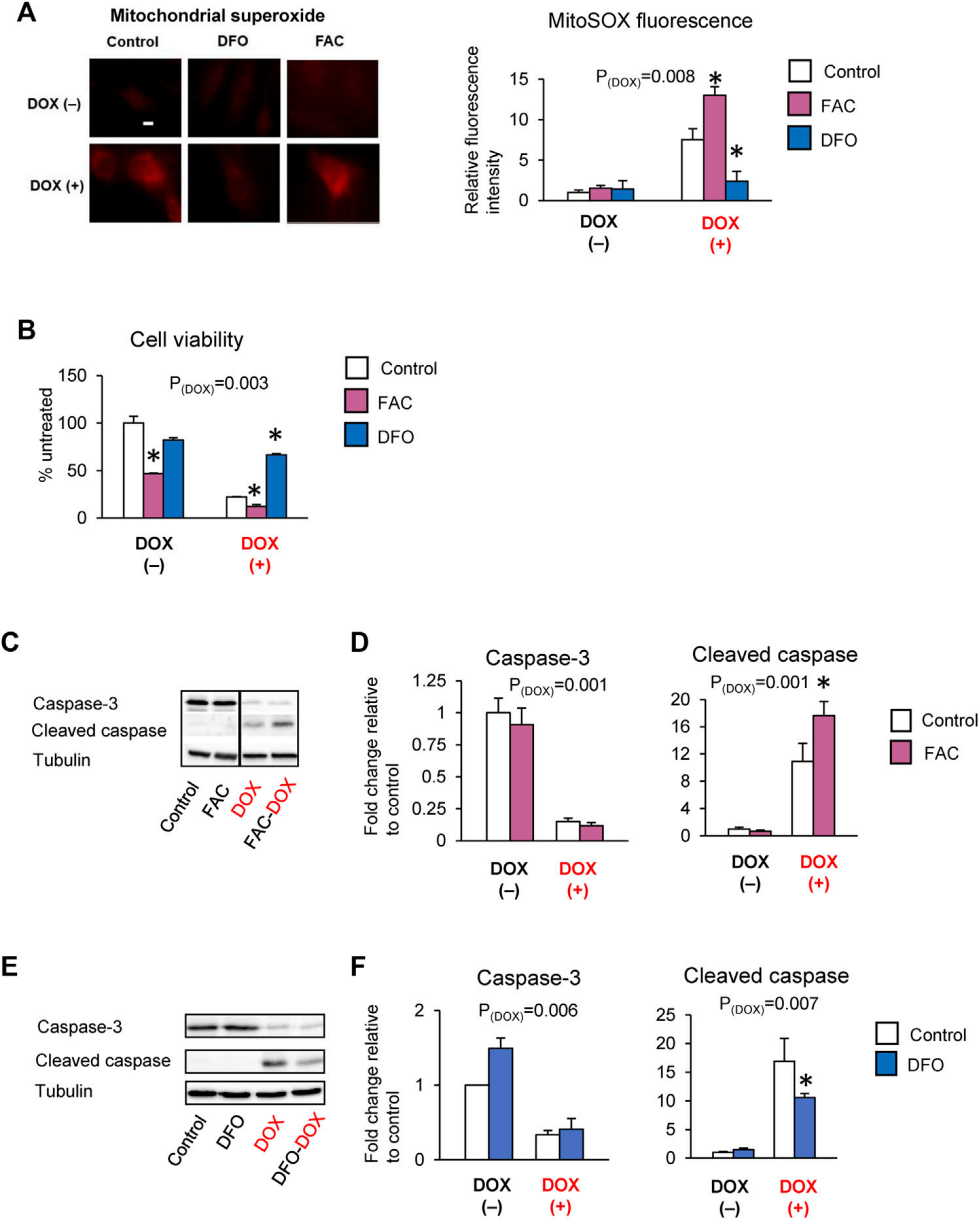
Iron exacerbates DOX cardiotoxicity. H9C2 cells were treated with ferric ammonium citrate (FAC) or deferoxamine (DFO) and DOX (10 µM) as indicated in the Methods section. ROS levels were analyzed by MitoSOX fluorescence **(A)**. Cell viability was measured using the MTT assay **(B)**. Caspase-3 and cleaved caspase levels were determined by western blot analysis **(C–F)**. Data were expressed as mean ± SEM. Statistical significance was assessed using the Student’s *t*-test or ANOVA with Tukey’s post-hoc comparisons. *p < .05 vs. control. Scale bar = 10 µm.

Studies have reported that DOX cardiotoxicity is associated with iron overload and that iron potentiates DOX-induced oxidative stress (Fang et al., 2019). Our findings that ABCB8 alters DOX retention and toxicity led us to question if iron regulates ABCB8 expression and thereby contributes to DOX accumulation. To delineate the role of iron in DOX-mediated ABCB8 depletion, we measured ABCB8 expression in cardiomyocytes with altered iron levels (Supplementary Figure S2). We found that ABCB8 mRNA and protein expression in cardiomyocytes was decreased upon iron overload and increased by iron depletion although no differences in mitochondrial iron were observed (Supplementary Figures S2A–D). In addition, ABCB8 downregulation caused by DOX treatment was exacerbated by iron overload and rescued by iron deficiency (Figures 3A,B). Consistent with ABCB8 expression, DOX retention was increased in iron-loaded cardiomyocytes, whereas iron deficiency dramatically decreased cardiac DOX retention (Figures 3C–E). Moreover, DOX-induced mitochondrial oxidative stress and cardiomyocyte death were aggravated by iron overload and corrected by iron deficiency (Figures 4A–F). Interestingly, FAC or DFO treatment did not alter mitochondrial iron levels (Supplementary Figure S2A) or DOX-induced mitochondrial iron overload (Supplementary Figure S2E). These results suggest that the effects of iron on DOX cardiotoxicity occur through ABCB8 rather than the redox potential of iron, which was previously thought.

To better understand the relationship between ABCB8 and iron status in DOX accumulation, we altered ABCB8 expression in iron-loaded and iron-deficient cardiomyocytes (Supplementary Figure S3 and Supplementary Figure S4). As expected, ABCB8-OE mitigated the inhibitory effects of iron overload on ABCB8 expression (Supplementary Figures S3A,B) and restored DOX efflux to levels similar to that of control (Supplementary Figures S3C–E). Conversely, knockdown of ABCB8 in iron-loaded cardiomyocytes resulted in almost complete depletion of ABCB8 protein (Supplementary Figures S3A,B) and prevented DOX efflux (Supplementary Figures S3C–E). Consequently, DOX toxicity, which was exacerbated by iron overload, was improved in ABCB8-OE and further worsened in ABCB8-KD (Supplementary Figures S3F–I). ABCB8 siRNA abrogated the effects of iron deficiency on DOX retention and toxicity (Supplementary Figures S4A–I), whereas DFO-treated ABCB8-OE cells displayed ABCB8 expression comparable to that of untreated controls and thus had lowest DOX retention and toxicity (Supplementary Figures S4A–I). Cumulatively, our data demonstrate that iron primarily facilitates DOX retention and exacerbates DOX cardiotoxicity by downregulating the efflux transporter, ABCB8.

### Cardiac DOX Retention and Toxicity are Exacerbated in a Mouse Model of HH

**FIGURE 5:**
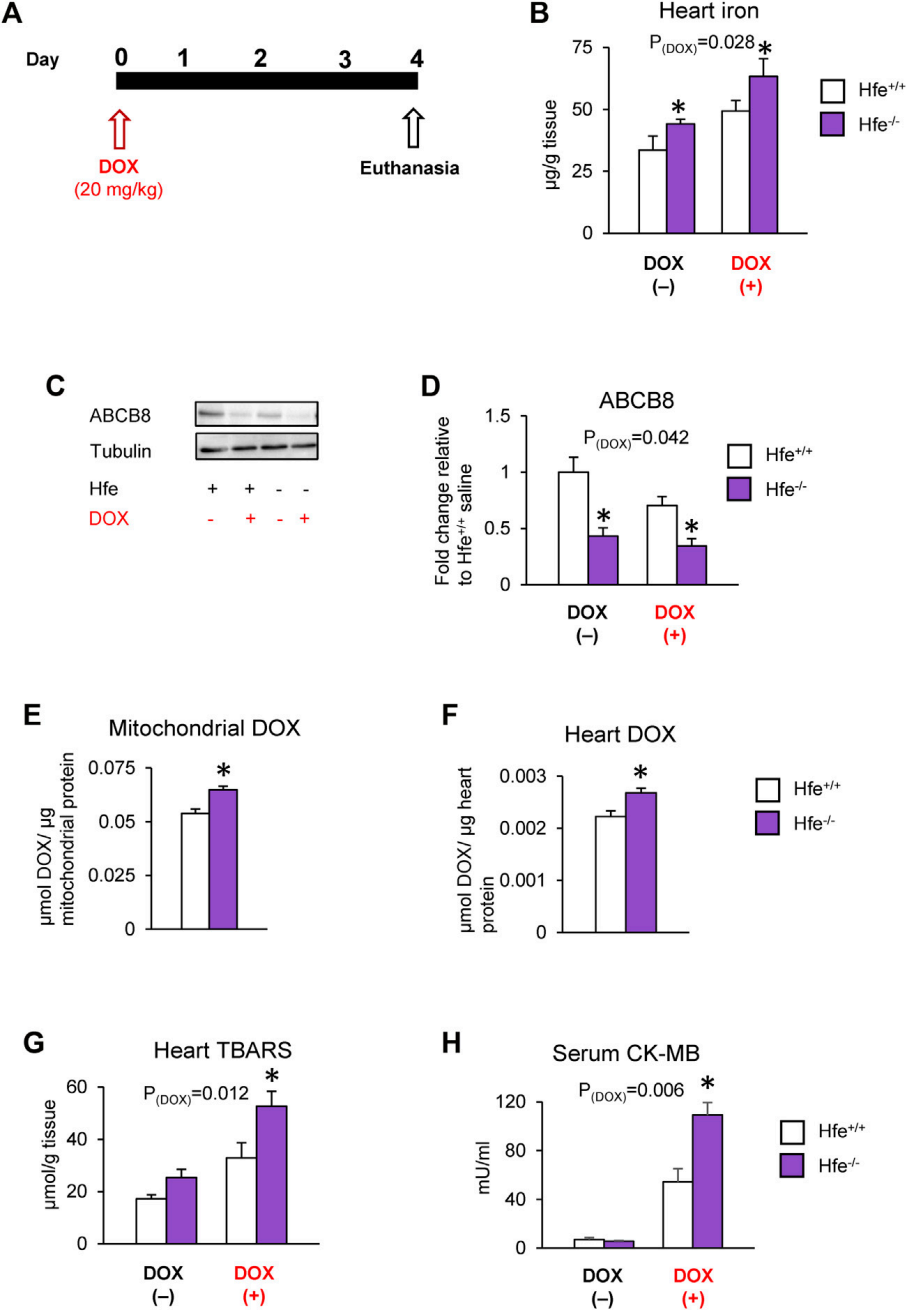
Cardiac DOX retention and toxicity are exacerbated in mice with iron overload hereditary hemochromatosis. Hfe-deficient (Hfe^−/−^) and control wild-type (Hfe^+/+^) mice were treated with DOX (20 mg/kg) as described in the Methods section **(A)**. Cardiac non-heme iron levels were measured by colorimetric assay using bathophenanthroline disulfonate **(B)**. ABCB8 levels in the heart were measured by western blot analysis and normalized to levels of *α*-tubulin **(C,D)**. DOX levels were measured in cardiac mitochondria **(E)** and cardiac tissue (**F**) by fluorescence and normalized to protein content. Oxidative stress was determined as TBARS levels and measured by colorimetric assay **(G)**. Serum CK-MB levels were measured by ELISA **(H)**. Results are representative of *n* = 6–7/group for all panels except **(C)** where *n* = 5/group. Data were expressed as mean ± SEM. Statistical significance was assessed using two-way ANOVA for 4-group comparisons with Tukey’s post-hoc comparisons and Student’s *t*-test for two-group comparisons. *p < .05 vs. Hfe^+/+^ of the same treatment.

We further tested our hypothesis *in vivo* using Hfe-deficient (Hfe^−/−^) mice, a mouse model of genetic iron overload (hereditary hemochromatosis; HH), after DOX treatment (Figure 5A). As reported previously (Miranda et al., 2003; Sukumaran et al., 2017), cardiac iron levels were elevated by acute DOX treatment and in hemochromatosis and were highest in DOX-treated Hfe^−/−^ mice (Figure 5B). Since iron is implicated in DOX cardiotoxicity through redox reactions (Gianni et al., 1985), we measured levels of labile (redox-active) iron as well as cardiac ferritin, the major iron storage protein. Interestingly, in parallel with iron overload, cardiac ferritin levels were also increased in HH or after DOX treatment (Supplementary Figure S5A), and there were no differences in cardiac labile iron levels among any groups (Supplementary Figure S5B).

We also found that both HH and DOX exposure decreased cardiac ABCB8 levels, which were lowest in DOX-treated Hfe^−/−^ mice (Figures 5C,D). While DOX increased mitochondrial iron, there was no difference in mitochondrial iron levels between DOX-exposed wild-type (Hfe^+/+^) and Hfe^−/−^ mice (Supplementary Figure S5C). However, Hfe^−/−^ mice exhibited increased DOX accumulation in the whole heart as well as in the mitochondria compared with Hfe^+/+^ mice (Figures 5E,F), whereas there was no difference in cytosolic DOX between Hfe^−/−^ and Hfe^+/+^ mice (Supplementary Figure S5D). Consequently, DOX-treated Hfe^−/−^ mice showed greater cardiac oxidative stress than Hfe^+/+^ mice (Figure 5G). Finally, DOX increased the levels of serum CK-MB, a marker of cardiotoxicity, which were dramatically higher in Hfe^−/−^ mice compared with Hfe^+/+^ mice (Figure 5H). Together, these results demonstrate that exacerbated DOX cardiotoxicity in iron overload conditions like HH result from increased cardiac DOX accumulation, rather than increased labile iron.

### Iron-Deficient Diet Decreases Cardiac DOX Accumulation and Toxicity in Mice

**FIGURE 6:**
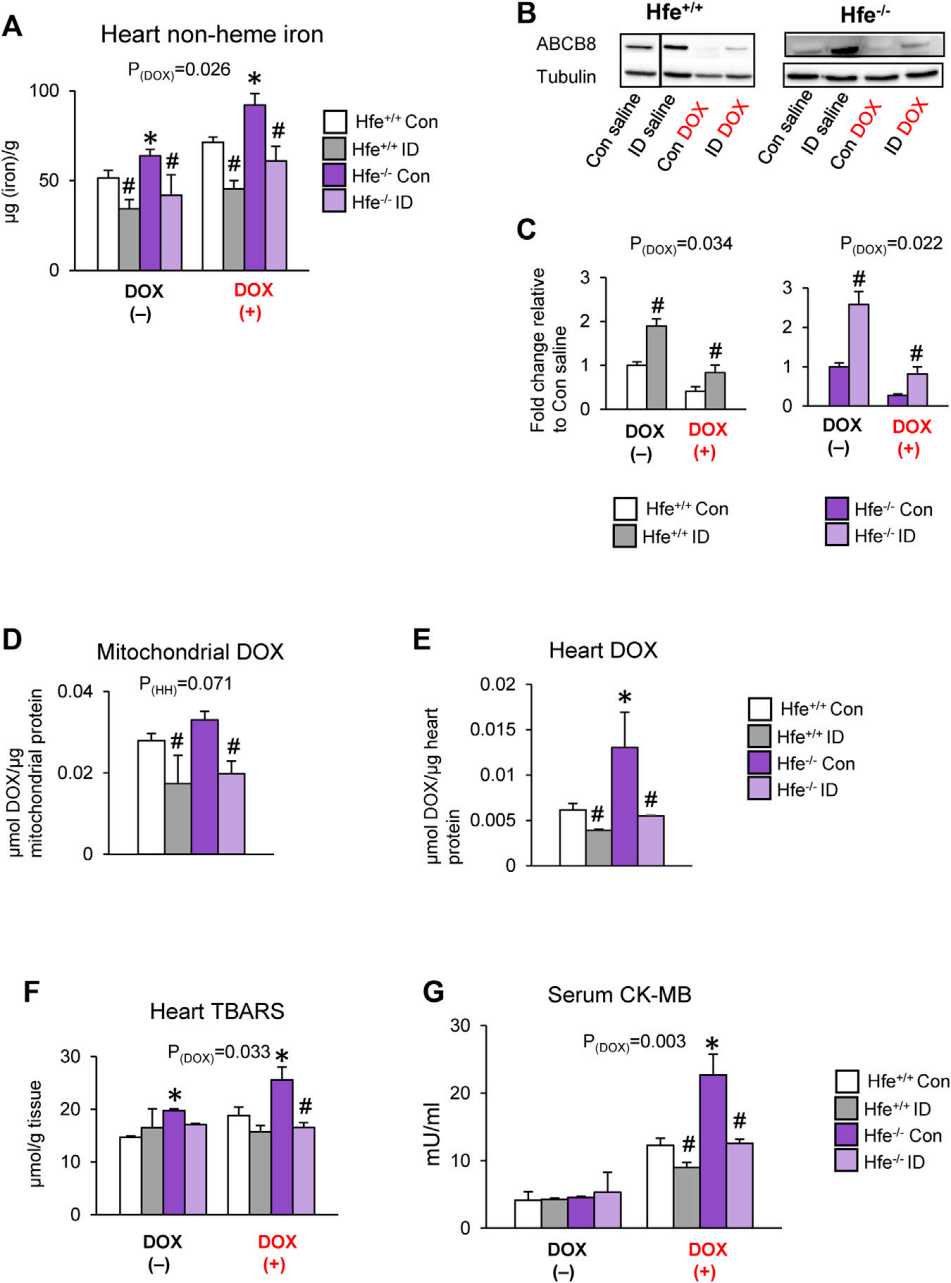
Correcting iron overload prevents exacerbated DOX cardiotoxicity in HH. Hfe^+/+^ and Hfe^−/−^ mice were maintained on iron-deficient (ID) diet and administered DOX (20 mg/kg) as described in the Methods section. Cardiac non-heme iron levels were measured by colorimetric assay using bathophenanthroline disulfonate **(A)**. ABCB8 levels in the heart were measured by western blot analysis and normalized to levels of *α*-tubulin **(B,C)**. Discontinuities between non-adjacent lanes of the same membrane were indicated by a solid line. Mitochondrial **(D)** and cardiac **(E)** DOX levels were measured in cardiac tissue by fluorescence and normalized to protein content. Oxidative stress was determined as TBARS levels and measured by colorimetric assay **(F)**. Serum CK-MB levels were measured by ELISA **(G)**. Results are representative of *n* = 7,8/group except **(B)** where *n* = 4,5/group. Data were expressed as mean ± SEM. Statistical significance was assessed using ANOVA with Tukey’s post-hoc comparisons. *p < .05 vs. Hfe^+/+^ of the same treatment. ^#^ *p* < .05 vs. control diet of the same genotype.

To confirm the effects of iron on cardiac DOX retention and toxicity, we maintained Hfe^+/+^ and Hfe^−/−^ mice on an iron-deficient (ID) diet. When serum iron levels in ID-fed Hfe^−/−^ mice were similar to those in Hfe^+/+^ mice on facility chow (Supplementary Figure S6A), i.e., when iron overload was corrected in Hfe^−/−^ mice, we induced acute DOX cardiotoxicity. The ID diet reduced cardiac iron levels in both genotypes, and ID-fed Hfe^−/−^ had cardiac iron comparable to facility chow-fed Hfe^+/+^ mice (Figure 6A). Also, ID prevented DOX-induced cardiac iron overload in both Hfe^+/+^ and Hfe^−/−^ mice (Figure 6A). Next, we determined if cardiac ABCB8 expression is modulated by ID. ABCB8 levels were increased in ID-fed mice compared with mice fed facility chow (control diet) regardless of genotypes (Figures 6B,C). ID also prevented DOX-induced ABCB8 depletion (Figures 6B,C) with no changes in mitochondrial iron levels (Supplementary Figure S6B). ID diet also reversed increased DOX accumulation in cardiac mitochondria and heart of Hfe^−/−^ mice (Figures 6D,E). Consequently, ID decreased cardiac oxidative stress and serum CK-MB levels in DOX-treated Hfe^+/+^ mice, and these beneficial effects were more pronounced in DOX-treated Hfe^−/−^ mice (Figures 6F,G). Collectively, our data demonstrate that iron overload (in HH) or deficiency (by diet) alters cardiac ABCB8 expression and thereby DOX retention and toxicity without affecting mitochondrial iron levels.

### Cardiac-Specific ABCB8 Overexpression Promotes DOX Efflux From Cardiac Mitochondria and Prevents DOX-Induced Cardiotoxicity

**FIGURE 7:**
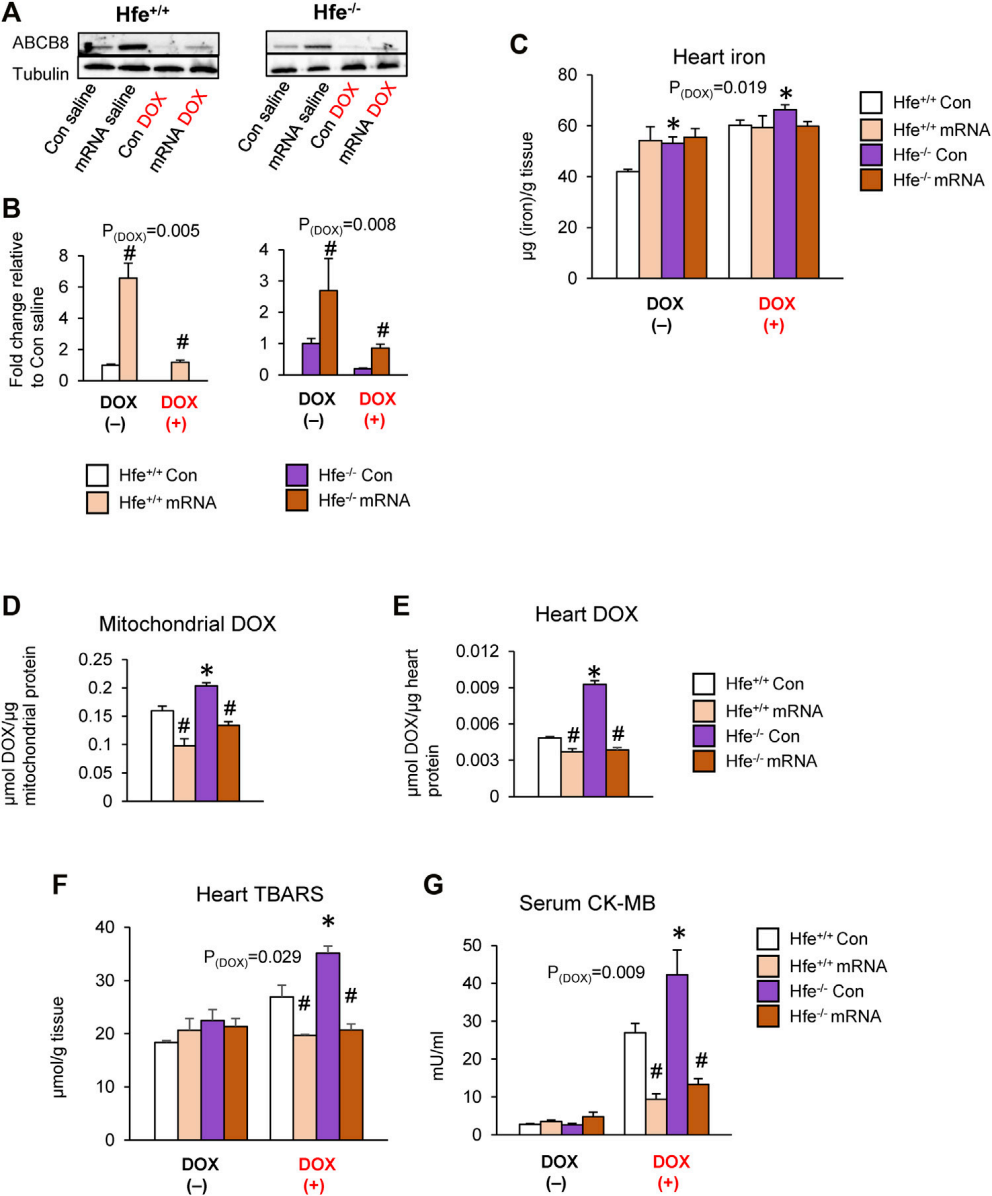
Intracardiac ABCB8 mRNA injection prevents mitochondrial DOX retention and cardiotoxicity despite cardiac iron overload. Hfe^+/+^ and Hfe^−/−^ mice received ABCB8 mRNA (15 µg) and DOX (20 mg/kg) as described in the Methods section. ABCB8 expression was determined by western blot analysis and normalized to *α*-tubulin levels **(A,B)**. Cardiac non-heme iron levels were measured by colorimetric assay using bathophenanthroline disulfonate **(C)**. Mitochondrial **(D)** and cardiac **(E)** DOX levels were measured in cardiac tissue by fluorescence and normalized to protein content. Oxidative stress was determined as TBARS levels and measured by colorimetric assay **(F)**. Serum CK-MB levels were measured by ELISA **(G)**. Results are representative of n = 6–7/group except **(A)** where n = 4-5/group. Data were expressed as mean ± SEM. Statistical significance was analyzed by ANOVA followed by Tukey’s post-hoc comparisons. *p < .05 vs. Hfe^+/+^ of the same treatment. ^#^ *p* < .05 vs. mRNA control of the same genotype.

To directly examine if DOX retention and toxicity are reversed by ABCB8 even in iron overload conditions, we upregulated cardiac ABCB8 levels by intracardiac delivery of ABCB8 mRNA. Injection of ABCB8 mRNA directly into the heart myocardium resulted in cardiac-specific overexpression of ABCB8 with no off-target effects (Supplementary Figures S7A,B). Although cardiac mitochondrial iron was insignificantly decreased (Supplementary Figure S7C), no differences were observed in tissue or mitochondrial iron levels in the kidney, liver, or lungs (Supplementary Figure S7C,D). We then determined the effects of ABCB8 overexpression on DOX retention and cardiotoxicity in HH mice. Intracardiac ABCB8 mRNA injection mitigated the deleterious effects of DOX on cardiac ABCB8 expression in both Hfe^+/+^ and Hfe^−/−^ mice (Figures 7A,B) with no influence on DOX-mediated cardiac iron overload (Figure 7C). Interestingly, ABCB8 overexpression decreased, although insignificantly, mitochondrial iron accumulation induced by DOX (Supplementary Figure S7E). Increased cardiac ABCB8 expression also decreased DOX retention in the mitochondria and cardiac tissue of Hfe^+/+^ and prevented DOX accumulation in Hfe^−/−^ mice without altering cytosolic DOX concentrations (Figures 7D,E, Supplementary Figure S7F). Congruently, DOX-mediated oxidative stress and cardiotoxicity were decreased in mice with ABCB8 overexpression (Figures 7F,G). Collectively, our results demonstrate that iron potentiates DOX cardiotoxicity through ABCB8 downregulation, and increased ABCB8 expression abrogates DOX cardiotoxicity even in iron overload conditions.

## Discussion

The anticancer agent DOX preferentially localizes in the mitochondria due to its affinity for the mitochondrial phospholipid, cardiolipin (de Wolf, 1991; Ichikawa et al., 2014). The mitochondrial exporter, ABCB8, is frequently up-regulated in DOX-resistant cancers, and correcting ABCB8 expression restores sensitivity to DOX (Gillet et al., 2004; Elliott and Al-Hajj, 2009; Basu et al., 2017; Wen et al., 2018). Conversely, DOX cardiotoxicity is associated with ABCB8 depletion (Ichikawa et al., 2014). In the current study, we found that intracellular DOX retention is potentiated in ABCB8-deficient cardiomyocytes and decreased in ABCB8 overexpression. Since DOX is metabolized to its semiquinone to produce ROS [reviewed in (Songbo et al., 2019)], increased DOX retention in ABCB8-KD cells would aggravate oxidative stress, whereas ABCB8 overexpression mitigates these effects. We note that our results are not congruent with those by Ichikawa et al., who reported that DOX retention does not differ in cardiomyocytes with altered ABCB8 expression (Ichikawa et al., 2014). This could be due to different experimental settings; Ichikawa et al. determined DOX concentrations under equilibrium conditions at one time point (16 h) (Ichikawa et al., 2014), whereas we employed a comprehensive kinetic approach to determine cellular DOX retention over time. ABCB8 is also implicated in mitochondrial iron efflux (Ichikawa et al., 2012). Consistent with previous results (Ichikawa et al., 2012; Ichikawa et al., 2014), we demonstrate that mitochondrial iron accumulation is potentiated by DOX and corrected by ABCB8 overexpression, although ABCB8-KD does not significantly induce mitochondrial iron accumulation. Since DOX itself has a high affinity for iron (Myers et al., 1982), it remains to be addressed if these changes in mitochondrial iron result from localization of DOX-iron complexes and if ABCB8 can facilitate the efflux of DOX-iron complexes. Combined, our data support the idea that, in addition to mitochondrial iron efflux, ABCB8 regulates the susceptibility to DOX cardiotoxicity through modifying its retention in cardiomyocytes.

In DOX-resistant cancers, which are associated with ABCB8 overexpression, cellular iron levels are decreased (Chekhun et al., 2013). Conversely, DOX cardiotoxicity is associated with cardiac iron overload and ABCB8 depletion (Miranda et al., 2003; Ichikawa et al., 2014). Accordingly, it has been known that DOX cardiotoxicity is potentiated by iron overload (Miranda et al., 2003; Panjrath et al., 2007), although this claim has been disputed (Guenancia et al., 2015), and the role of iron in DOX cardiotoxicity remains unknown. Additionally, other events, such as disruption of the DNA repair enzyme Top2β and calcium homeostasis dysregulation (Fu et al., 1990; Zhang et al., 2012), have been implicated as the causative mechanisms underlying DOX cardiotoxicity. In the present study, we sought to define the role of iron in DOX cardiotoxicity by altering cellular iron status by pretreatment with FAC or DFO and found that iron downregulates ABCB8 expression. Ichikawa et al. demonstrated that ABCB8 regulates mitochondrial iron homeostasis by exporting iron from the mitochondria (Ichikawa et al., 2012). Importantly, our studies revealed that, altering cellular iron status (by FAC or DFO) does not change mitochondrial iron levels despite differences in ABCB8 expression. These results suggest that exacerbated DOX cardiotoxicity in iron overload does not result from disruption of mitochondrial iron homeostasis and that compensatory mechanisms may exist to override the role of ABCB8 in regulating mitochondrial iron. These findings were also corroborated by our *in vivo* results and previous studies by Jouihan et al., who reported no differences in mitochondrial iron levels in mice with HH (Jouihan et al., 2008). While mitochondrial iron levels were unchanged, DOX retention and, therefore, toxicity were increased in iron-loaded cardiomyocytes and decreased in iron deficiency. Together, our data affirm that elevated intracellular iron depletes ABCB8 and augments DOX accumulation, thereby increasing DOX cardiotoxicity.

Lipshultz et al. reported exacerbated cardiac dysfunction in HH carriers as compared with non-carriers (Lipshultz et al., 2013). Mouse models of HH also demonstrate increased mitochondrial damage in acute DOX cardiotoxicity (Miranda et al., 2003). While iron overload was observed in DOX treatment and HH, the iron storage protein ferritin was also upregulated likely by adaptive response, and this could explain why cardiac labile (redox) iron levels were unchanged in the DOX + HH group. However, DOX-associated oxidative stress in HH was still greater. Increased DOX retention is associated with higher oxidative stress (Mai et al., 2016), which may result from iron-independent mechanisms, such as induction of ROS-producing enzymes or DOX-semiquinone production (Songbo et al., 2019). Previous studies have demonstrated that increased DOX accumulation, either by deficiency of DOX efflux transporters [ABCB1 (Asperen et al., 1999)] or by co-administration with drugs that increase tissue DOX accumulation (Colombo et al., 1994), increases the toxic effects of DOX. Thus, while our results support the previous finding that HH exacerbates DOX cardiotoxicity, we offer a novel mechanism by which iron overload downregulates ABCB8 expression and consequently increases DOX retention.

HH is primarily characterized by iron overload, but other factors including the Hfe gene and the iron regulatory hormone, hepcidin, are also perturbed, and these factors have been thought to play an important role in DOX cardiotoxicity (Levy et al., 1999; Miranda et al., 2003; Cocco et al., 2013; Rockley, 2018). To delineate the role of iron, we normalized elevated iron status in Hfe^−/−^ mice by feeding them an ID diet. Although studies have reported that dietary iron levels alter the susceptibility to DOX cardiotoxicity, the exact mechanisms are yet to be identified (Panjrath et al., 2007; Fang et al., 2019). We found that ID diet prevents DOX-induced iron overload and ABCB8 depletion and therefore decreases DOX retention and toxicity. Together, our results validate that exacerbated DOX cardiotoxicity in HH results from iron overload, rather than hepcidin or Hfe gene deficiency.

Another important question in our study was whether correction of ABCB8 expression that was downregulated in HH would protect against DOX cardiotoxicity. Changes in iron stores in the heart are less dynamic than those in other organs like the liver (Sukumaran et al., 2017), and therefore modifying cardiac iron may not be a timely strategy to prevent DOX cardiotoxicity in cancer patients. RNA therapies have been explored in the treatment of cardiovascular dysfunctions (Lesizza et al., 2017; Carlsson et al., 2018) although ABCB8 mRNAs have not been evaluated. Our approach resulted in cardiac-specific overexpression of ABCB8, which corrected DOX accumulation and toxicity despite iron overload. Our findings suggest a novel therapeutic strategy to circumvent DOX toxicity and preserve cardiac function. Although we utilized mRNA as a mechanistic tool, further improvements to ensure mRNA uptake only by cardiomyocytes or pharmacological intervention using drugs that can upregulate ABCB8 expression, as well as detailed analyses of off-target effects, may increase the therapeutic utility of these approaches.

Taken together, our studies identify two novel mechanisms involved in DOX cardiotoxicity; we demonstrate that ABCB8 plays an important role in DOX efflux and that ABCB8 expression is regulated by iron status. Follow-up studies are required to identify if ABCB8 levels are regulated by cellular (as well as subcellular) or systemic iron status. Our findings that FAC and DFO alter both cardiac ABCB8 expression and intracellular iron (without modifying mitochondrial iron) provide an important insight that ABCB8 levels might not be regulated by mitochondrial iron. In addition, the exact mechanism of substrate mobilization by ABCB8 remains to be determined. While no differences in DOX uptake were observed in our studies, the role of iron or ABCB8 in cardiolipin expression or activity is largely unestablished (Liu et al., 1990; Faust, 2017). Also, why DOX reduces iron in cancer resistant cells (Chekhun et al., 2013) but increases cardiac iron levels remains to be addressed. Although studies suggest that DOX alters iron homeostasis in cancer cells and cardiomyocytes through estrogen response (Pokrzywinski et al., 2018; Bajbouj et al., 2019), the exact mechanism remains to be defined. Our study provides an improved understanding of the mechanisms by which iron plays a key role in exacerbating DOX cardiotoxicity in iron overload conditions such as HH.

## Data Availability

The raw data supporting the conclusion of this article will be made available by the authors, without undue reservation.

## Abbreviations

ABCB8, ATP binding cassette subfamily B8; DFO, deferoxamine; DOX, doxorubicin; FAC, ferric ammonium citrate; HFE, hyperferremia; HH, hereditary hemochromatosis.
