# Supplementary material for: Iron Promotes Cardiac Doxorubicin Retention and Toxicity Through Downregulation of the Mitochondrial Exporter ABCB8
Source: Front Pharmacol. 2022 Mar 11;13:817951. doi: 10.3389/fphar.2022.817951 (PMC8963208; doi:10.3389/fphar.2022.817951)

SUPPLEMENTARY MATERIAL

SUPPLEMENTARY FIGURE LEGENDS

Supplementary Figure S1. ABCB8 expression does not alter mitochondrial iron levels in DOX-treated cardiomyocytes. H9C2 cells were transfected with targeted siRNA or plasmid DNA to knockdown (ABCB8 KD) **(A, B)** or overexpress (ABCB8 OE) **(C, D)** ABCB8, respectively. RPA and calcein fluorescence was normalized to Hoechst fluorescence **(E, F)**. Results are representative of 4 independent experiments. Data were expressed as mean ± SEM. Statistical significance was assessed using the Student's *t*-test. * $p < 0.05$ vs. control. Scale bar = 10 µm.

Supplementary Figure S2. Iron exposure alters the levels of cytosolic iron, but not mitochondrial iron. H9C2 cells were treated with ferric ammonium citrate (FAC), deferoxamine (DFO) as described in the Methods section. RPA and calcein fluorescence was normalized to Hoechst fluorescence **(A)**. ABCB8 mRNA **(B)** and protein **(C, D)** levels were measured using qRT-PCR and western blotting, respectively. Cells were treated with DOX (10 µM) for 24 h. RPA and calcein fluorescence was normalized to Hoechst fluorescence. **(E)**. Results are representative of 4 independent experiments. Data were expressed as mean ± SEM. Statistical significance was assessed using the Student's *t*-test. * $p < 0.05$ vs. control. Scale bar = 10 µm.

Supplementary Figure S3. Iron overload promotes DOX retention and toxicity through ABCB8 downregulation. H9C2 cells were transfected with siRNA or plasmid DNA and maintained in media containing FAC as described in the Methods section. Cells were treated with DOX (10 µM) for 24 h. ABCB8 protein expression was determined by western blot analysis and normalized to α-tubulin levels **(A, B)**. Discontinuities between non-adjacent lanes from the same blot were indicated by solid black lines. After 48 h of FAC treatment, cells were incubated with DOX (30 µM) for 3 h and fluorescence from live cells was measured at the indicated times. Results are representative of 4 independent experiments **(C)**. DOX fluorescence was quantified over time **(D)**. The area under the curve (AUC) was calculated **(E)** from the DOX retention plot **(D)**. ROS levels were analyzed by MitoSOX fluorescence **(F)**. Cell viability was measured using the MTT assay **(G)**. Levels of caspase-3 and its cleaved form were determined as apoptosis markers by western blot analysis **(H, I)**. Data were expressed as mean ± SEM. Statistical significance was

assessed ANOVA with Tukey's post-hoc comparisons. * $p < 0.05$ vs. DOX alone. $^{\#}$ $p < 0.05$ vs DOX-FAC in cells with altered ABCB8 expression. Scale bar = 10 µm.

Supplementary Figure S4. Iron deficiency decreases DOX retention and toxicity through ABCB8 upregulation. H9C2 cells were transfected with siRNA or plasmid DNA and maintained in media containing DFO (200 µM) as indicated in the Methods section. ABCB8 protein expression was determined using western blot and normalized to α-tubulin levels **(A, B)**. Discontinuities between non-adjacent lanes from the same blot were indicated by solid black lines. After 48 h of DFO treatment, cells were incubated with DOX (30 µM) for 3 h. Results are representative of 4 independent experiments **(C)**. DOX fluorescence was quantified over time and normalized to Hoechst **(D)**. AUC was determined from the DOX retention plot **(E)**. ROS levels were analyzed by MitoSOX fluorescence **(F)**. Cell viability was measured using the MTT assay **(G)**. Levels of caspase-3 and its cleaved form were determined as apoptosis markers by western blot analysis **(H, I)**. Data were expressed as mean ± SEM. Statistical significance was assessed using ANOVA with Tukey's post-hoc comparisons. * $p < 0.05$ vs. DOX alone. $^{\#}$ $p < 0.05$ vs DOX-DFO in cells with altered ABCB8 expression. Scale bar = 10 µm.

Supplementary Figure S5. Exacerbated DOX cardiotoxicity in iron overload hemochromatosis does not result from perturbations in iron homeostasis. Mice received DOX (20 mg/kg) as described in the Methods section. Cardiac ferritin levels were measured by ELISA **(A)**. Labile iron levels were measured in the heart by fluorescence assay (Ex/Em: 485/538) using dihydrorhodamine **(B)**. Non-heme iron levels in cardiac mitochondria were measured by colorimetric assay using bathophenanthroline disulfonate **(C)**. DOX levels were quantified in cytosolic fractions using fluorescence and normalized to cytosolic protein content (**D**). Results are representative of n=6-7/group. Data were expressed as mean ± SEM. Statistical significance was assessed using two-way ANOVA with Tukey's post-hoc comparisons for 4-group comparisons and the Student's *t*-test for two-group comparisons. * $p < 0.05$ vs $Hfe^{+/+}$ of the same treatment.

Supplementary Figure S6. Exacerbated DOX cardiotoxicity in iron overload hemochromatosis is corrected by low-iron diet. $Hfe^{+/+}$ and $Hfe^{-/-}$ mice were maintained on iron-deficient (ID) diet for 8 weeks to correct cardiac iron overload **(A)**. Non-heme iron levels in cardiac mitochondria were measured by colorimetric assay using bathophenanthroline disulfonate **(B)**. DOX levels were quantified in cytosolic fractions using fluorescence and normalized to cytosolic protein content (**C**). Results are representative of n=7-8/group. Data were expressed as mean ±

SEM. Statistical significance was assessed using ANOVA with Tukey's post-hoc comparisons. * $p < 0.05$ vs $Hfe^{+/+}$ of the same treatment. # $p < 0.05$ vs control diet of the same genotype.

Supplementary Figure S7. Intracardiac ABCB8 mRNA injection results in cardiac-specific ABCB8 overexpression. ABCB8 mRNA (15 µg) was administered as described in the Methods section. ABCB8 expression was determined by western blot and normalized to α-tubulin levels **(A, B)**. Non-heme iron levels were measured in mitochondria **(C, E)** and tissues **(D)** by colorimetric assay using bathophenanthroline disulfonate. DOX levels were quantified in cytosolic fractions using fluorescence and normalized to cytosolic protein content (**F**). Results are representative of n=4/group (A-D) or 6-7/group (E-F). Data were expressed as mean ± SEM. Statistical significance was assessed using the Student's *t*-test (A-D) or ANOVA with Tukey's post-hoc comparisons (E-F). * $p < 0.05$ vs. $Hfe^{+/+}$ of the same treatment.

Supplementary Figure S1

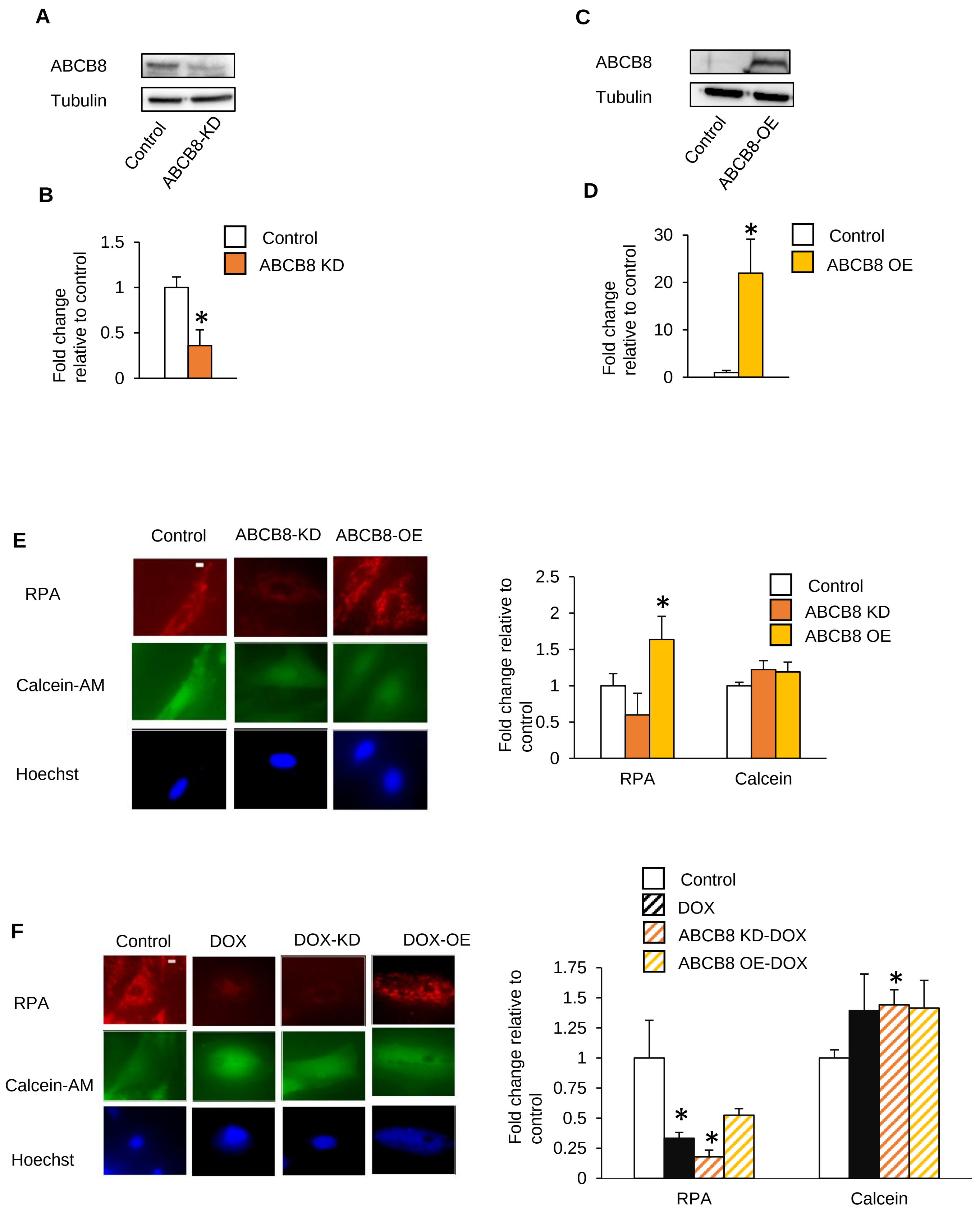

Supplementary Figure S2

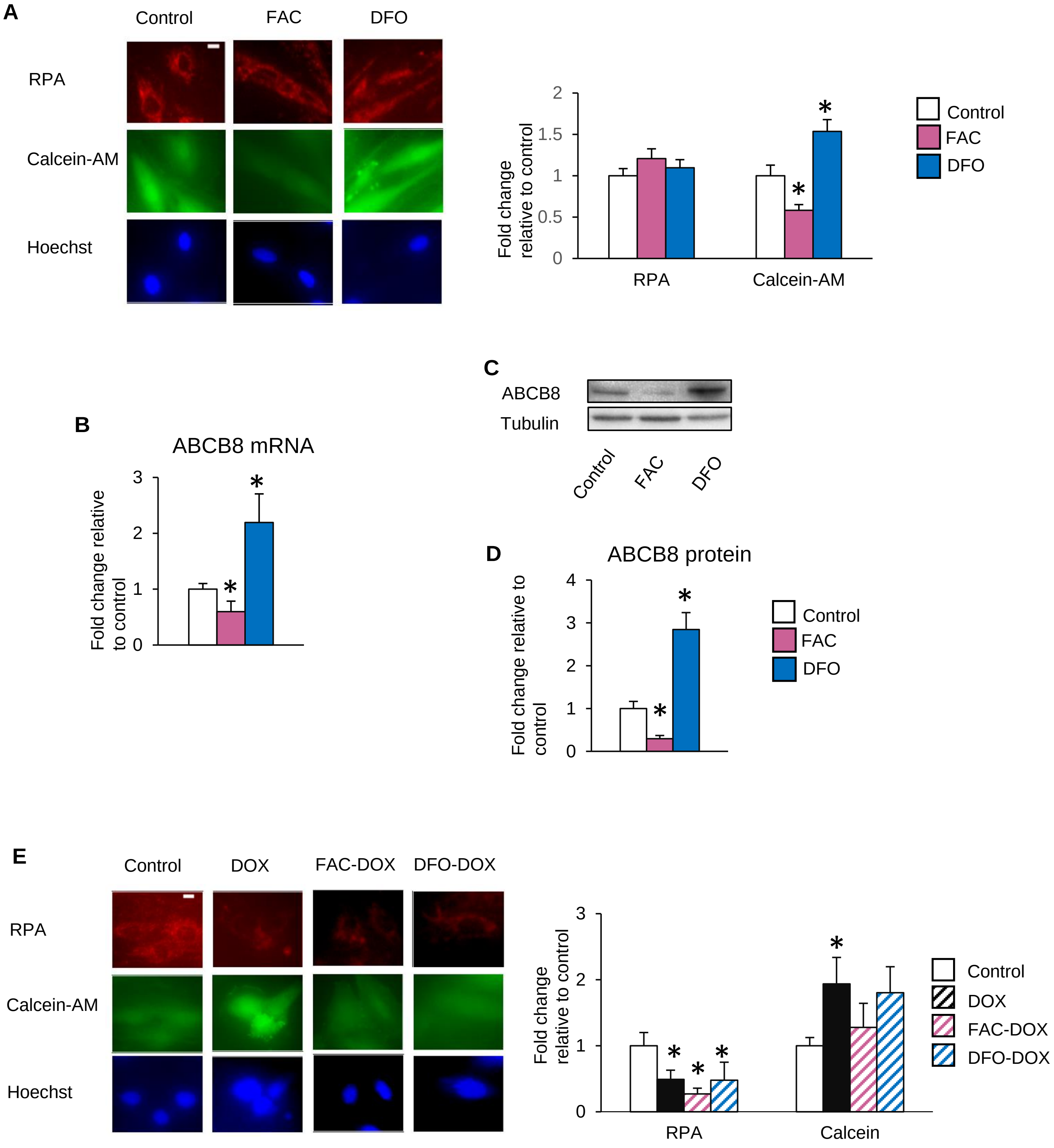

Supplementary Figure S3

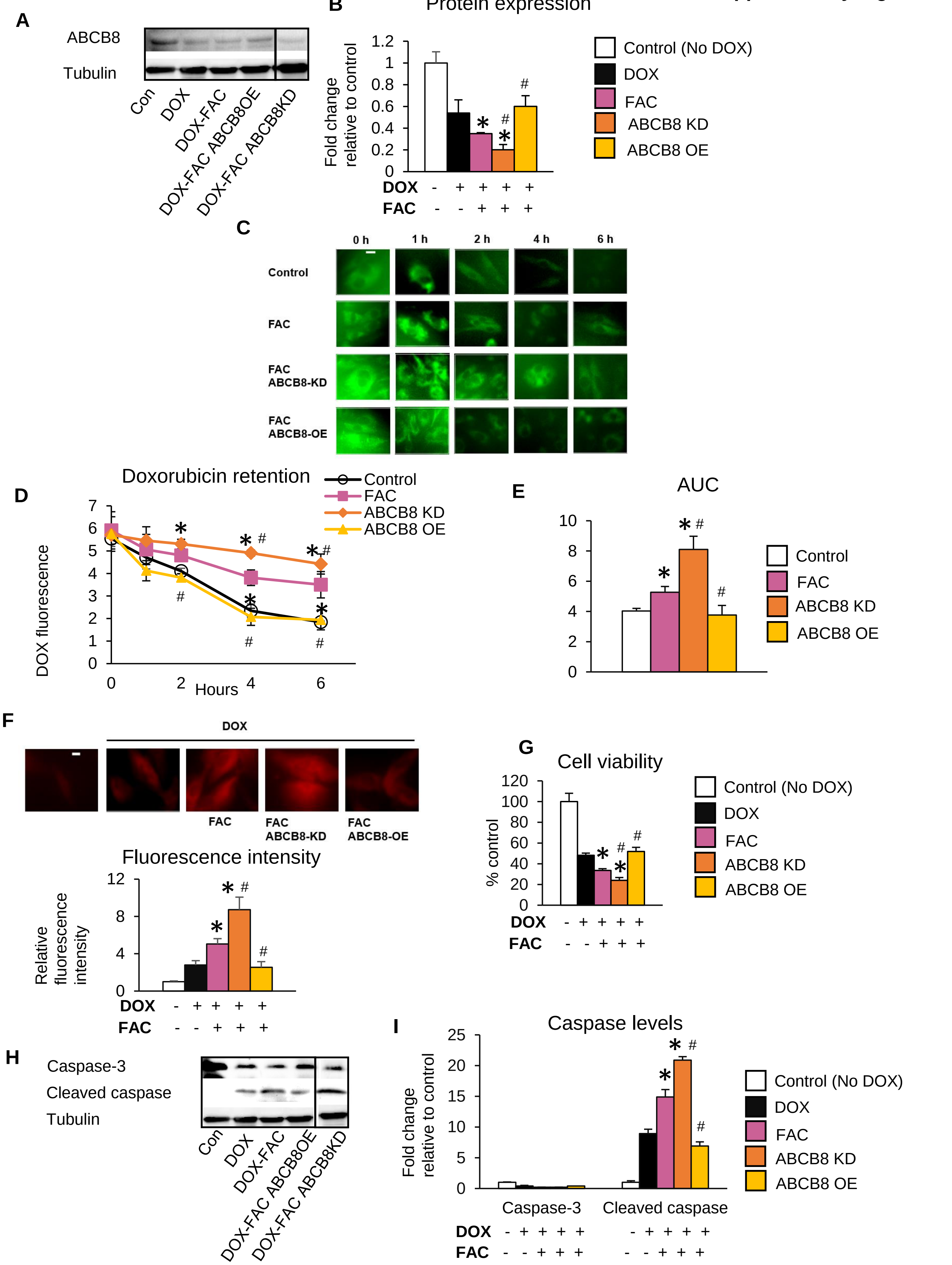

Supplementary Figure S4

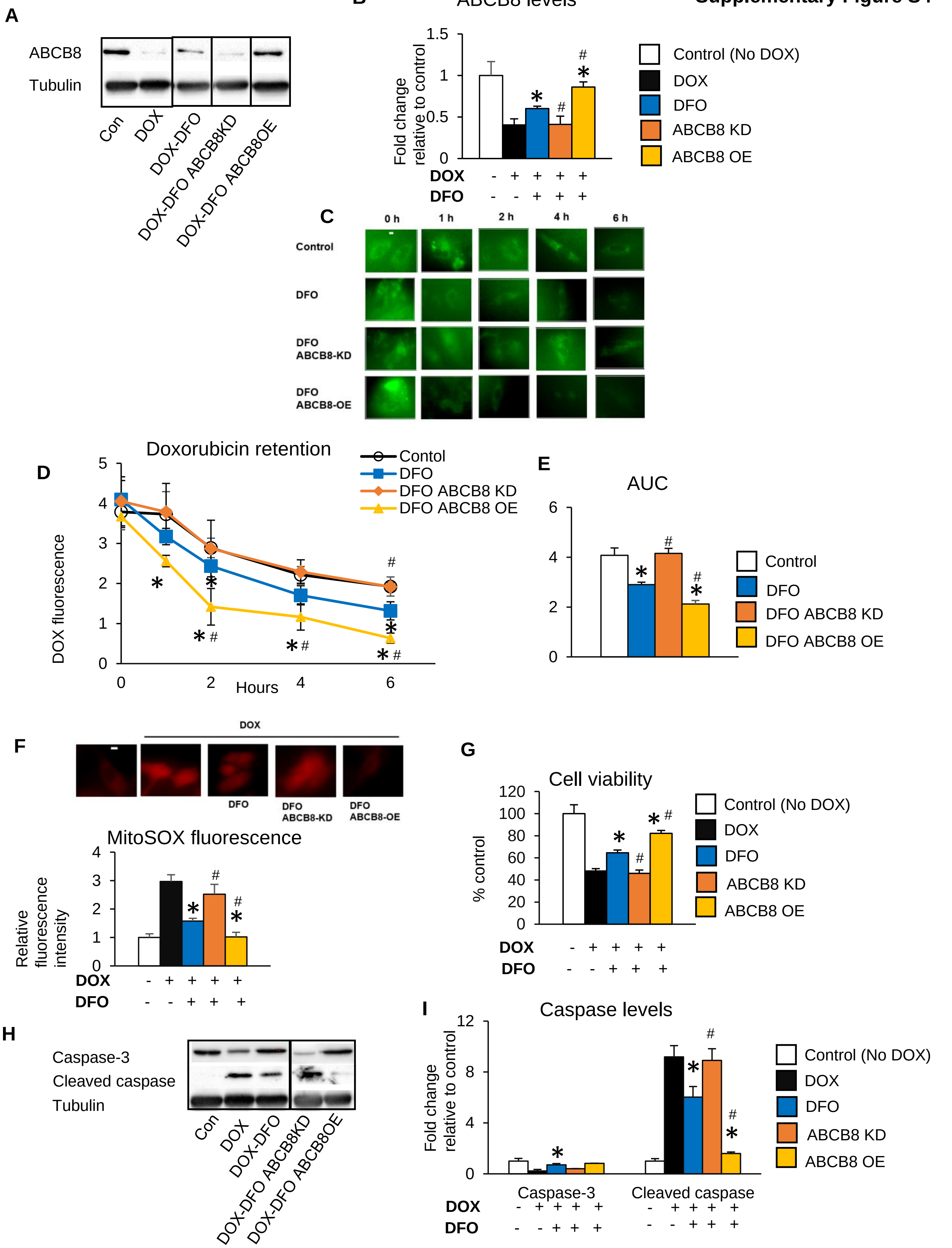

Supplementary Figure S5

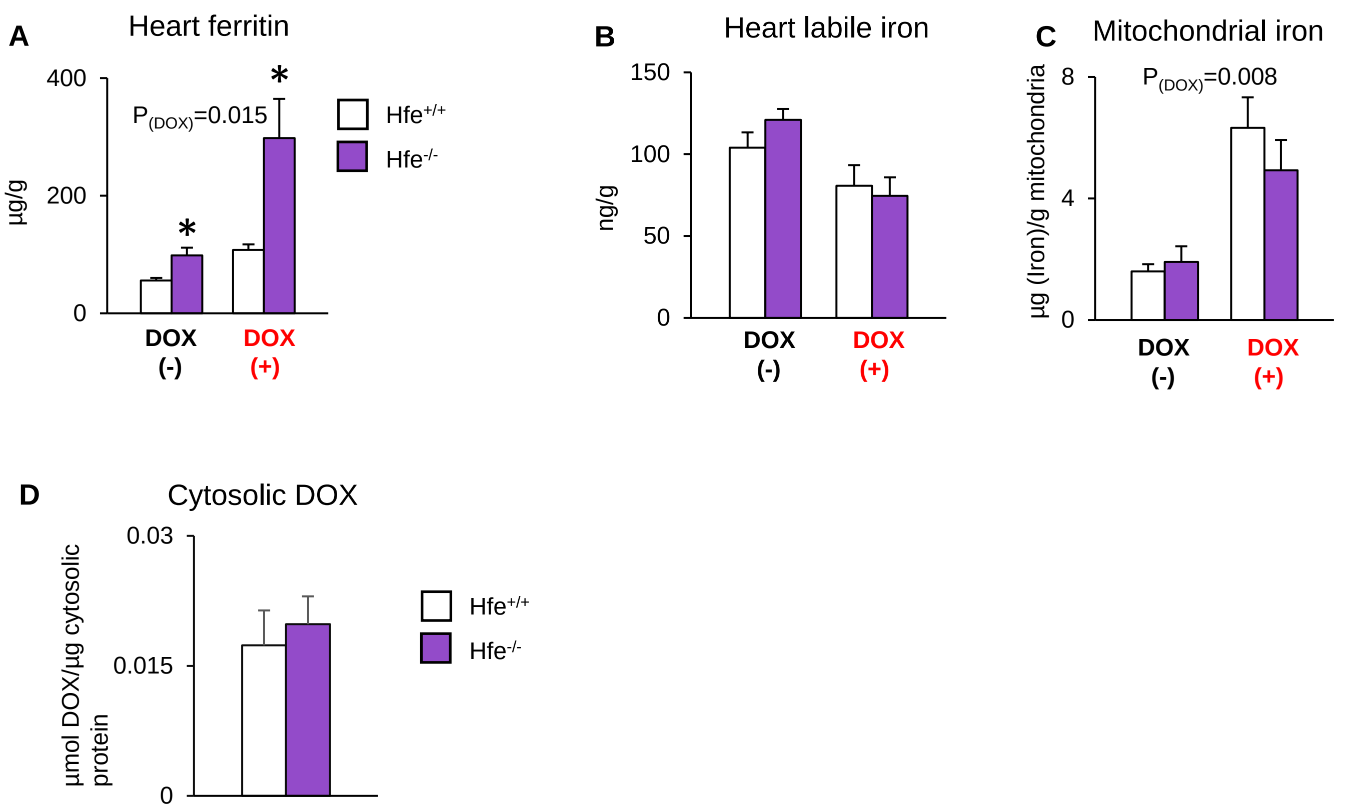

Supplementary Figure S6

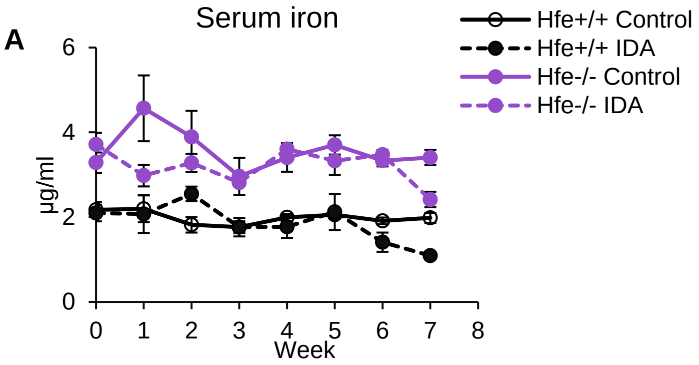

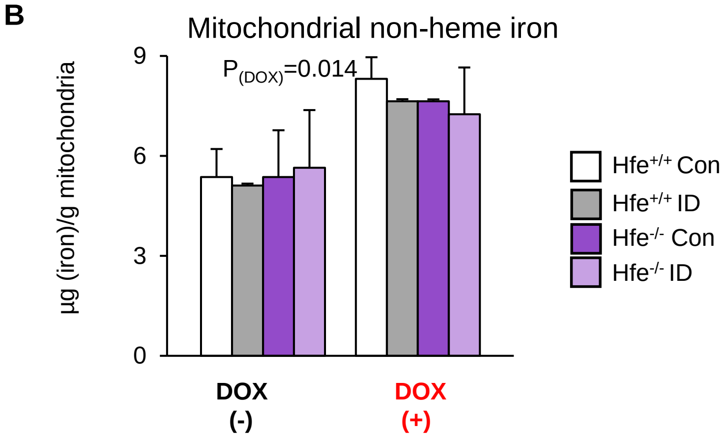

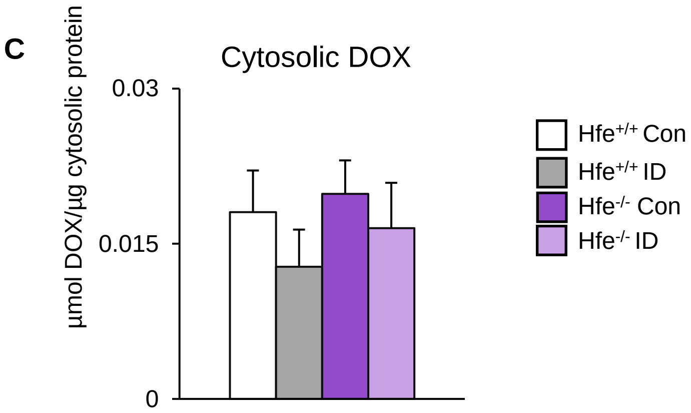

Supplementary Figure S7

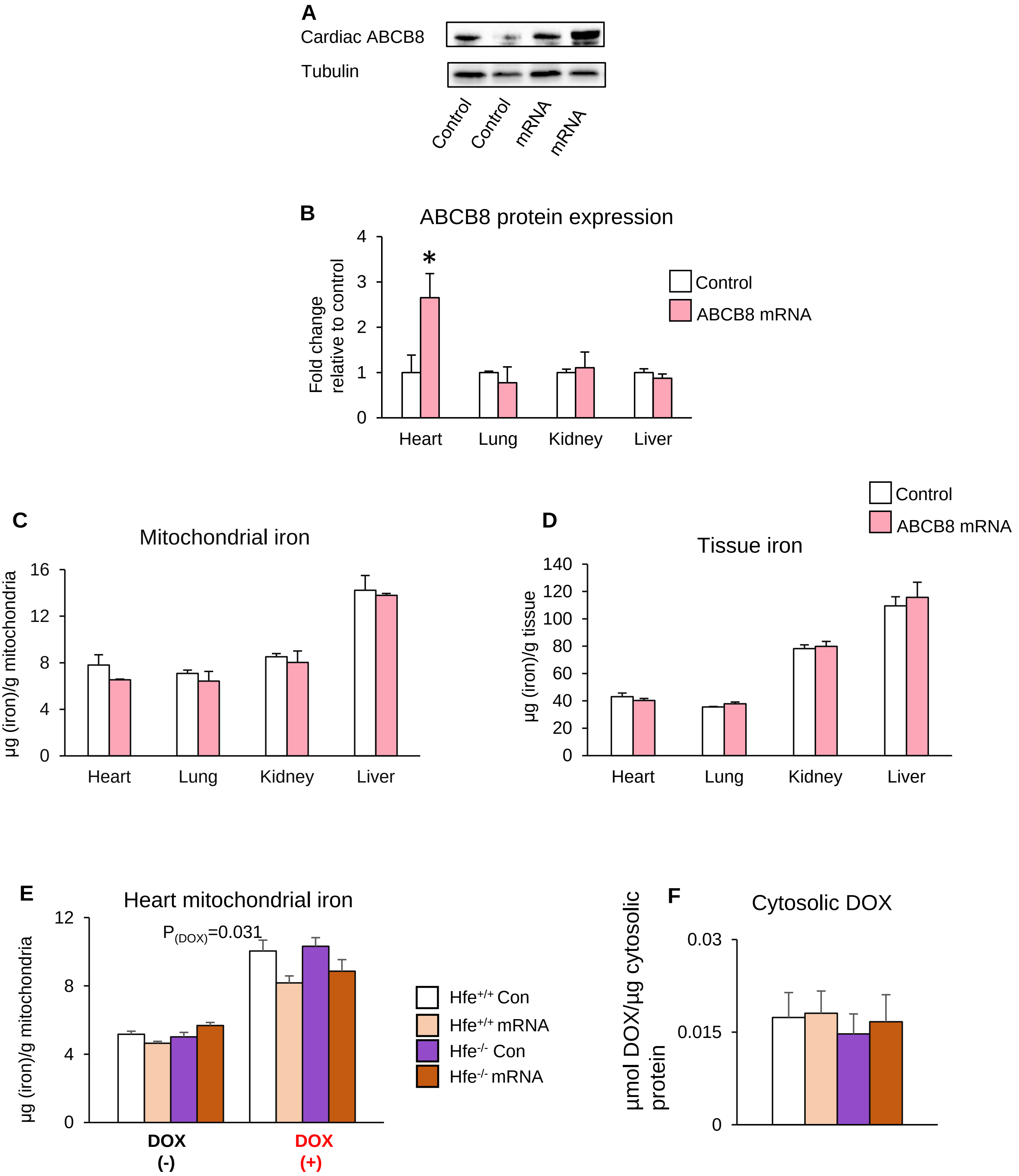

Supplement: Supplementary file 1 [file Presentation1.pdf]
